# Association of smoking and right ventricular function in middle age: CARDIA study

**DOI:** 10.1136/openhrt-2020-001270

**Published:** 2020-03-08

**Authors:** Henrique T Moreira, Anderson C Armstrong, Chike C Nwabuo, Henrique D Vasconcellos, Andre Schmidt, Ravi K Sharma, Bharath Ambale-Venkatesh, Mohammad R Ostovaneh, Catarina I Kiefe, Cora E Lewis, Pamela J Schreiner, Stephen Sidney, Kofo O Ogunyankin, Samuel S Gidding, Joao A C Lima

**Affiliations:** 1 Division of Cardiology, Johns Hopkins University, Baltimore, Maryland, USA; 2 Division of Cardiology, University of Sao Paulo, Ribeirao Preto, Sao Paulo, Brazil; 3 Population and Quantitative Health Sciences, University of Massachusetts Medical School, Worcester, Massachusetts, USA; 4 Division of Preventive Medicine, University of Alabama at Birmingham, Birmingham, Alabama, USA; 5 Division of Epidemiology and Community Health, University of Minnesota, Minneapolis, Minnesota, USA; 6 Division of Research, Kaiser Permanente Division of Research, Oakland, California, USA; 7 Division of Cardiology, Northwestern University Feinberg School of Medicine, Chicago, Illinois, USA; 8 Chief Medical Officer, The FH Foundation, Passadena, California, USA

**Keywords:** echocardiography, smoking, cardiac function

## Abstract

**Objective:**

To evaluate the association of cigarette smoking and right ventricular (RV) systolic and diastolic functions in a population-based cohort of individuals at middle age.

**Methods:**

This cross-sectional study included participants who answered the smoking questionnaire and underwent echocardiography at the Coronary Artery Risk Development in Young Adulthood year 25 examination. RV systolic function was assessed by echocardiographic-derived tricuspid annular plane systolic excursion (TAPSE) and by right ventricular peak systolic velocity (RVS’), while RV diastolic function was evaluated by early right ventricular tissue velocity (RVE’). Multivariable linear regression models assessed the relationship of smoking with RV function, adjusting for age, sex, race, body mass index, systolic blood pressure, total cholesterol, high-density lipoprotein (HDL) cholesterol, diabetes mellitus, alcohol consumption, pulmonary function, left ventricular systolic and diastolic function and coronary artery calcium score.

**Results:**

A total of 3424 participants were included. The mean age was 50±4 years; 57% were female; and 53% were black. There were 2106 (61%) never smokers, 750 (22%) former smokers and 589 (17%) current smokers. In the multivariable analysis, current smokers had significantly lower TAPSE (β=−0.082, SE=0.031, p=0.008), RVS’ (β=−0.343, SE=0.156, p=0.028) and RVE’ (β=−0.715, SE=0.195, p<0.001) compared with never smokers. Former smokers had a significantly lower RVE’ compared with never smokers (β=−0.414, SE=0.162, p=0.011), whereas no significant difference in RV systolic function was found between former smokers and never smokers.

**Conclusions:**

In a large multicenter community-based biracial cohort of middle-aged individuals, smoking was independently related to both worse RV systolic and diastolic functions.

**Key questions**

**What is already known about this subject?**

- Smoking is a major cardiovascular risk factor.

**What does this study add?**

- Smoking is independently related to both worse right ventricular systolic and diastolic functions.

**How might this impact on clinical practice?**

- Stopping smoking might prevent right heart failure.

## Introduction

Smoking is a major cardiovascular risk factor. The relationship between cigarette consumption and cardiovascular disease (CVD) is long-time recognised, with early description back to 1964 in the very first surgeon general’s report.^1^ After more than half of a century, strong evidence supports the association between tobacco use and multiple cardiovascular conditions, including coronary heart disease and stroke.^2^ Several mechanisms have been linked to smoking-related CVD, such as atherogenesis, platelet dysfunction, prothrombotic and antifibrinolytic effects, vasomotor dysfunction, inflammation and modification of lipid profile.^3^

Despite extensive epidemiological, clinical and experimental data explaining the association of cigarette consumption and harmful consequences on cardiovascular function, many aspects of this relationship remain unclear, in particular, the effect of smoking on right ventricular (RV) function. The right ventricle is potentially affected in smokers who develop severe chronic obstructive pulmonary disease (COPD) and pulmonary hypertension, although other pathways may be involved, such as left ventricular (LV) dysfunction, subclinical atherosclerosis and chronic systemic inflammatory response.

Thus, the aim of this study was to evaluate the association of cigarette smoking with RV systolic and diastolic functions in a population-based cohort of individuals at middle age.

## Methods

### Study participants

The Coronary Artery Risk Development in Young Adulthood (CARDIA) study is a prospective community-based cohort enrolling initially 5115 black and white men and women aged 18–30 years in 1985–1986 free of known CVD, originally designed to investigate the development of CVD and their risk factors. Participants were recruited and examined at four field centres: Birmingham, Alabama; Chicago, Illinois; Minneapolis, Minnesota; and Oakland, California. Of 3498 participants (72% of surviving cohort) attending the year 25 (2010–2011) examination, 3445 (98.5%) answered the smoking questionnaire. Of those who answered the smoking questionnaire, 3424 also underwent echocardiography and were included in this present investigation.

### Smoking

Smoking status was ascertained by structured questionnaires. Current smokers were those who smoked more than five cigarettes per day for more than 3 months, while former smokers were those who had previously smoked more than five cigarettes per day for more than 3 months but were not currently smoking. Never smokers were those who had never smoked or who had smoked more than five cigarettes/day for more than 3 months in their lifetime. For current smokers, cigarette consumption intensity and duration were assessed as number of cigarretes smoked/day, total years of smoking regularly and cigarrete pack-years (packs/day×years smoking).

### Clinical measurements

Participants were asked to avoid smoking or engaging in heavy physical activity for at least 2 hours before each examination and to fast for 12 hours.^4^ Systolic blood pressure (SBP) and diastolic blood pressure (DBP) were obtained 5 min after the participant had been seated, and the second and third readings were averaged. Blood samples were drawn to measure lipid profile, glucose level and glycated haemoglobin. Diabetes mellitus (DM) was defined as a fasting plasma glucose of ≥126 mg/dL, 2 hours postload glucose of ≥200 mg/dL during a 75 g oral glucose tolerance test, glycated haemoglobin of ≥6.5% or use of antidiabetes medication. Average daily consumption of alcohol was calculated from questionnaire information. Educational status was verified using standardised questionnaire and measured as years of education.

Pulmonary function testing was performed at CARDIA year 20 examination (2005–2006) using a dry rolling-seal OMI spirometer (Viasys Corporation, Loma Linda, California, USA), as previously described.^5^ Forced expiratory volume in one second (FEV_1_) and forced vital capacity were measured according to standard spirometer procedures as recommended by the American Thoracic Society.^6^

Coronary artery calcium (CAC) score was measured from CT by using 64-channel multidetector scanners (Siemens, Erlangen, Germany, or GE Healthcare, Milwaukee, Wisconsin, USA) at year 25 examination, as detailed elsewhere.^7^

### Echocardiography

The echocardiographic protocol at the CARDIA year 25 examination has been previously published.^8 9^ Briefly, transthoracic echocardiography was performed using an Artida ultrasound system (Toshiba Medical Systems, Tokyo, Japan), with phased-array transducers from 1.8 to 4.2 MHz, following a standardised acquisition protocol. Technicians at each of the four field centres were centrally trained and certified. Parasternal longitudinal and short-axis views, as well as apical four-chamber and two-chamber views, were acquired. Measurements of RV function were performed at the apical four-chamber view focused on the right ventricle.

RV systolic function was assessed using tissue Doppler-derived tricuspid annular peak systolic velocity (right ventricular peak systolic velocity (RVS’)). The higher the RVS’, the better the RV systolic function. RV systolic function was also examined by using M-mode-derived tricuspid annular plane systolic excursion (TAPSE). The higher the TAPSE, the better the RV systolic function. RV diastolic function was evaluated by tissue Doppler-derived early diastolic tricuspid annular velocity (right ventricular early diastolic velocity (RVE’)). The higher the RVE’, the better the RV diastolic function. Cut-off values for defining RV systolic and diastolic dysfunctions were based on the latest guidelines from the American Society of Echocardiography as follows: RVS’<0.95 cm/s, TAPSE <17 mm/s and RVE’<7.8 cm/s.^10^

LV volumes and LV ejection fraction were estimated by biplane Simpson’s method. LV mass was calculated from M-mode measures using the cube formula.^10^. LV longitudinal strain, a measure of myocardial systolic function, was derived from speckle tracking analysis, as previously described.^9^ LV diastolic function was assessed by tissue-Doppler derived LV lateral e’. Pulsed Doppler-derived E-wave from mitral inflow was used to derive the E/e’ ratio, an index of LV filling pressure. Left atrial volume was calculated using the disk summation algorithm in the apical four-chamber view. Pulmonary artery systolic pressure (PASP) was derived from tricuspid regurgitant jet velocity using the modified Bernoulli equation, as published elsewhere.^11^

### Statistical analysis

Continuous data are reported as mean±SD if normally distributed, and median (IQR) if non-normally distributed. Histograms and skewness test were performed to assess the normality of the data. The primary cigarette consumption exposure variable in this study was smoking status. In those who were current smokers at year 25, smoking intensity (number of cigarettes smoked per day), smoking duration (years of smoking regularly altogether) and cumulative smoking exposure (cigarette pack-years) were analysed by dividing into tertiles for an exploratory analysis. Differences in baseline characteristics across smoking status were evaluated by test for trend across ordered groups,^12^ Student t-test and χ^2^ test as appropriate. Multivariable linear regression models assessed the relationship of smoking status, smoking intensity, smoking duration and cumulative smoking exposure (independent variables) with RV systolic and diastolic functions (dependent variables), adjusting for age, sex, race, educational status, body mass index (BMI), SBP, total cholesterol, HDL cholesterol, DM, antihypertensive and lipid-lowering medications, alcohol consumption, FEV_1_, LV ejection fraction, LV E/e’ ratio and CAC. Interaction terms were used to verify whether the association of smoking status with RV function is modified by sex and race. The variance inflation factor was used to test collinearity, with a mean value of 1.35, reflecting absence of significant multicollinearity. All tests were two-tailed. Values of p<0.05 were considered significant. All statistical analyses were performed with STATA V.15.1.

### Patient involvement

Participants were not involved in setting the research question or the outcome measures, nor were they involved in developing plans for recruitment, design or implementation of the study. No patients were asked for advice on interpretation or writing up of results. Results from the CARDIA study were disseminated to the community, including study participants, via newsletters and website (https://www.cardia.dopm.uab.edu).

## Results

A total of 3424 participants were included, with a mean age of 50±4 years; 57% were female; and 53% were black. Never smokers, former smokers and current smokers were 2106 (61%), 750 (22%), 589 (17%) individuals, respectively. Current smokers reported a median smoking intensity of 10 (IQR: 5–15) cigarettes/day, and a median duration of 29 (IQR: 20–34) years of smoking regularly, while median cumulative cigarette smoking exposure was 9.0 (IQR: 4.5–13.5) pack-years.

TAPSE, RVS’ and RVE’ were obtained in 3177 (93%), 3262 (95%) and 3282 (96%) participants included in this study. TAPSE, RVS’ and RVE’ were not feasible in 247 (7%), 162 (5%) and 182 (4%) individuals due to poor image quality. Mean TAPSE, RVS’ and RVE’ were 2.5±0.5 cm, 13.4±2.5 cm/s and 12.4±3.2 cm/s, respectively. Reduced RVS’, TAPSE and RVE’ were found in 119 (3.4%), 94 (2.5%) and 75 (2.2%) participants, respectively. Mean LV ejection fraction and LV lateral e’ were 61%±7% and 11.8±2.8 cm/s, respectively. Mean PASP was 31±6 mm Hg, measured in 1283 (38%) participants who had a significant tricuspid regurgitant jet.

Participants’ characteristics according to smoking status are presented in table 1. The proportions of women in never smoker, former smoker and current smoker status were 57%, 60% and 51% (p=0.007), respectively. A majority of current smokers were black, 62%, while the proportions of black race in never smoker and former smoker status were 46% and 36%, respectively (p<0.001).

**Table 1:** Characteristics of the participants according to smoking status

	Smoking status						
	N	F	C		Comparisons between groups†		
	n=2106	n=750	n=589	P value*	N versus F	N versus C	F versus C
Demographic and clinical parameters							
Age (years)	50.1±3.6	50.9±3.5	49.6±3.7	<0.001	<0.001	0.027	<0.001
Female	57%	60%	51%	0.007			
Black race	46%	36%	62%	<0.001			
Body mass index (kg/m^2^)	30±7	30±7	29±7	0.028	1.000	0.039	0.052
Education (years)	16 (14–18)	15 (13–16)	13 (12–15)	<0.001	<0.001	<0.001	<0.001
Systolic blood pressure (mm Hg)	119±16	118±15	123±17	<0.001	0.838	<0.001	<0.001
Diastolic blood pressure (mm Hg)	74±11	74±11	77±12	<0.001	1.000	<0.001	<0.001
On antihypertensive medication (%)	26	27	32	<0.001			
Total cholesterol (mg/dL)	192±36	193±37	193±41	0.781			
HDL cholesterol (m/dL)	58±17	59±18	56±20	0.017	0.194	0.281	0.013
On lipid-lowering medication (%)	15	17	16	0.331			
Diabetes mellitus (%)	13	13	17	0.098			
Alcohol consumption/day (mL)	2 (0–12)	5 (0–17)	8 (0–27)	<0.001	<0.001	<0.001	<0.001
Echocardiography							
RVS' (cm/s)	13.5±2.5	13.5±2.6	13.2±2.5	0.038	0.768	0.031	0.112
TAPSE (mm)	2.56±0.50	2.57±0.53	2.45±0.50	<0.001	1.000	<0.001	<0.001
RVe' (cm/s)	12.7±3.3	12.3±3.0	11.8±3.0	<0.001	0.024	<0.001	0.027
LV end-diastolic volume (mL)	112±31	110±31	113±30	0.192			
LV end-systolic volume (mL)	44±18	43±20	45±18	0.169			
LV ejection fraction (%)	61±7	62±7	61±7	0.092			
LV mass index (g/m^2^)	82±21	84±21	89±22	<0.001	0.021	<0.001	<0.001
LV longitudinal strain (%)	−15.1±2.4	−15.3±2.4	−14.7±2.3	<0.001	0.489	0.001	<0.001
E wave (cm/s)	79±16	78±17	79±17	0.465			
LV lateral wall e' (cm/s)	11.9±2.9	11.7±2.8	11.2–2.8	<0.001	0.656	<0.001	0.004
LV E/e'	7.0±2.3	7.0±2.3	7.6±2.9	<0.001	1.000	<0.001	<0.001
LA volume index (mL/m^2^)	24.5±6.9	24.5±7.0	25.0±7.2	0.343			
PASP (mm Hg)	31.1±5.7	30.8±5.6	32.1±7.7	0.794			
TAPSE/PASP (mm/mm Hg)	0.84 (0.71–0.98)	0.84 (0.68–1.00)	0.76 (0.64–0.94)	0.003	0.985	<0.001	0.005
Pulmonary function							
FEV_1_ (L)	3.1±0.8	3.1±0.8	2.9±0.8	<0.001	1.000	<0.001	0.001
FVC (L)	3.9±1.0	4.0±1.0	3.8±1.0	0.006	1.000	0.019	0.007
FEV_1_/FVC	0.80 (0.76–0.84)	0.78 (0.75–0.82)	0.78 (0.73–0.82)	<0.001	<0.001	<0.001	0.255
CAC score (>0 AU)	24%	30%	41%	<0.001			

Continuous variables are expressed as mean±SD if normally distributed and as median (IQR) if non-normally distributed; categorical variables are reported as counts (%).

*Test for trend for continuous variables, and χ^2^ test for categorical variables.

†Student t-test or Wilcoxon rank-sum test.

AU, Agatston unit; C, current; CAC, coronary artery calcium; E/e', Ratio of pulsed-Doppler derived E-wave from mitral inflow to tissue-Doppler derived left ventricular lateral e'; F, former; FEV_1_, forced expiratory volume in one second; FVC, forced vital capacity; HDL, high-density lipoprotein; LA, left atrial; LV, left ventricular; N, never; PASP, pulmonary artery systolic pressure; RVe', right ventricular early diastolic velocity; RVS', right ventricular peak systolic velocity; TAPSE, tricuspid annular plane systolic excursion.

RVS’ was significantly worse in current smokers (13.2±2.5 cm/s) compared with never smokers (13.5±2.5 cm/s), but not significantly different between current smokers and former smokers. When assessed by TAPSE, RV systolic function was also significantly worse in current smokers (2.45±0.5 cm), in comparison with both never smokers (2.56±0.53 cm) and former smokers (2.57±0.53 cm), p<0.001 for both comparisons, but not significantly different between never and former smokers. RV diastolic function evaluated by RVE’ was significantly worse in current smokers (11.8±3.0 cm/s) in comparison with both never smokers (12.7±3.3 cm/s) (p<0.001), and former smokers (12.3±3.0 cm/s) (p=0.027). RVE’ was significantly lower in formers smokers in comparison with never smokers (12.7±3.3 cm/s) (p=0.024).

Educational status decreased, while daily alcohol consumption and proportion of positive CAC score increased from those who had never smoked towards current smokers. Current smokers had higher SBP, higher DBP and lower FEV_1_ compared with both never smokers and former smokers. BMI was significantly lower in current smokers in comparison with those who had never smoked, while HDL cholesterol was significantly lower in current smokers in comparison with former smokers.

### Multivariable analysis

In the multivariable analysis, current smokers showed a reduced RVS’ (β=−0.343, SE=0.156, p=0.028) and a reduced TAPSE (β=−0.082, SE=0.031, p=0.008) in comparison with never smokers, independently of age, sex, race, educational status, traditional cardiovascular risk factors, alcohol consumption, pulmonary function, LV systolic and diastolic function and CAC score (tables 2 and 3).

**Table 2:** Linear regression analyses for the association of smoking status with RV systolic function assessed by tricuspid annular peak systolic velocity (RVS')

	Univariate			Model 1		
	β	SE	P value	β	SE	P value
Smoking status						
Never (ref.)	Ref.	Ref.	Ref.	Ref.	Ref.	Ref.
Former	−0.033	0.111	0.768	−0.114	0.130	0.383
Current	−0.263	0.122	0.031	−0.343	0.156	0.028

β=coefficient; model 1: adjusted for age, sex, race, years of education, body mass index, systolic blood pressure, use of antihypertensive medication, total cholesterol, high-density lipoprotein cholesterol, use of lipid-lowering medication, forced expiratory volume in one second, left ventricular ejection fraction, pulsed-Doppler derived mitral E-wave/tissue-Doppler derived left ventricular lateral e’ ratio and coronary artery calcium score.

ref., reference; RV, right ventricular; RVS', right ventricular peak systolic velocity.

**Table 3:** Linear regression analyses for the association of smoking status with RV systolic function assessed by tricuspid annular plane systolic excursion (TAPSE)

	Univariate			Model 1		
	β	SE	P value	β	SE	P value
Smoking status						
Never (ref.)	Ref.	Ref.	Ref.	Ref.	Ref.	Ref.
Former	0.005	0.023	0.819	−0.023	0.026	0.376
Current	−0.115	0.025	<0.001	−0.082	0.031	0.008

β=coefficient; model 1: adjusted for age, sex, race, years of education, body mass index, systolic blood pressure, use of antihypertensive medication, total cholesterol, high-density lipoprotein cholesterol, use of lipid-lowering medication and forced expiratory volume in one second, left ventricular ejection fraction, pulsed-Doppler dervied mitral E-wave/tissue-Doppler derived left ventricular lateral e’ ratio and coronary artery calcium score.

ref., reference; RV, right ventricular.

Compared with never smokers, both current smokers and former smokers had significantly lower RVE’ in the multivariable model (β=−0.715, SE=0.195, p<0.001, for former smokers, and β=−0.414, SE=0.162, p=0.011, for current smokers) (table 4).

**Table 4:** Linear regression analyses for the association of smoking status with RV diastolic function assessed by early diastolic tricuspid annular tissue velocity (RVE’)

	Univariate			Model 1		
	β	SE	P value	β	SE	P value
Smoking status						
Never (ref.)	Ref.	Ref.	Ref.	Ref.	Ref.	Ref.
Former	−0.371	0.140	0.008	−0.414	0.162	0.011
Current	−0.847	0.155	<0.001	−0.715	0.195	<0.001

β=coefficient; model 1: adjusted for age, sex, race, years of education, body mass index, systolic blood pressure, use of antihypertensive medication, total cholesterol, high-density lipoprotein cholesterol, use of lipid-lowering medication and forced expiratory volume in one second, left ventricular ejection fraction, pulsed-Doppler derived mitral E-wave/tissue-Doppler derived left ventricular lateral e’ ratio and coronary artery calcium score.

RV, right ventricular; RVS', right ventricular peak systolic velocity.

Detailed descriptions of the models are present in online supplementary tables 1-3.

10.1136/openhrt-2020-001270.supp1 Supplementary data

Race and sex showed no significant interaction with the association of smoking status and RV systolic and diastolic function.

### Exploratory analysis: smoking intensity, duration and pack-years

Smoking duration was characterised as years of smoking regularly by tertile: first tertile=less than 26 years; second tertile=from 26 to 32 years; and third tertile=equal or more than 33 years. Those in the third smoking duration tertile had worse TAPSE in comparison with those in the first tertile (β=−0.116, SE=0.051, p=0.024) in the univariate analysis, although this relationship was not significant in the fully adjusted model 1 (online supplementary table 4). Neither RVS’ nor RVE’ were different among smoking duration tertiles. Neither systolic nor diastolic RV functions were significantly related to smoking intensity or cigarette pack-years (online supplementary tables 5 and 6).

## Discussion

Since the initial reports warning the harmful effects of tobacco use, many investigations have studied the association of cigarette consumption with CVD, usually seeking conditions affecting the left ventricle.^1^ By contrast, our study is focused on the relationship between smoking and the right ventricle, performed in a large multicenter community-based biracial cohort of middle-aged individuals. Our findings demonstrate that smoking is associated with both worse RV systolic and diastolic function.

In our study, current smokers had worse RV systolic function than those who never smoked, independent of demographics, concurrent traditional cardiovascular risk factors, pulmonary function measured 5 years earlier, LV systolic and diastolic functions, and CAC score. In another large study from the Multi-Ethnic Study of Atherosclerosis (MESA), current smokers had both lower RV mass and lower RV volume as assessed by MRI.^13^ In contrast, smoking status was not associated with RV structure and function as examined by echocardiography in the Atherosclerotic Risk in Communities (ARIC) study. Of note, in the ARIC study, participants were elderly (mean age of 75.8±5.1 years); only 6.3% (n=287) were smokers; and many with smoking related events may have died before study analysis.^14^ In this present analysis of the CARDIA year 25 examination, participants were at middle age (50±4 years), and 17% (n=589) were current smokers.

We also demonstrated that not only current smokers but also former smokers had worse RV diastolic function compared with never smokers. Prior investigation has reported an acute effect of cigarette smoking on RV diastolic function as assessed by echocardiography.^15 16^ Smoking a single cigarette can rapidly lead to oxidative damage of endothelial cells, increases in heart rate and blood pressure, and coronary vasoconstriction.^17^ These acute changes in cardiovascular function might explain, at least in part, the association of current smoker status with worse RV diastolic function showed in our study. Nonetheless, according to our findings, former smokers also had worse RV diastolic function in comparison with never smokers, which may suggest a role of persistent harmful effects of smoking on the right ventricle. Interestingly, RV systolic function was not significantly different in these two groups, suggesting that different pathways may link smoking with RV systolic and diastolic function. Further research is required to examine differences in pathophysiological mechanisms on RV impairment associated with smoking.

In an exploratory analysis, we examined three components of smoking exposure in current smokers: intensity, duration and pack-years (product of intensity and duration). Our results showed that longer duration of continuous smoking might be related to worse RV systolic function, as evaluated by TAPSE. However, this result was inconsistent when RV systolic function was assessed by RVS’, which was not related to duration of smoking. Furthermore, the relationship between lower TAPSE and worse RV systolic function was not statistically significant in the multivariable model. No relationship between intensity and pack-years with RV systolic and diastolic function was found. A recent investigation from the MESA study found that smoking duration was not associated with CVD, while smoking intensity was a stronger correlate of CVD outcomes.^18^ Such diverse results might be explained by different pathophysiological pathways linking smoking to RV dysfunction compared with those related to smoking and other CVD. Also, smoking intensity and duration may vary over time, which may lead to imprecise measurements in self-reported questionnaires.

Although pulmonary vascular remodelling is usually present in patients with advanced COPD, it can also occur in those with mild COPD and even in smokers with normal lung function.^19 20^ Furthermore, RV systolic function, hypertrophy and dilation can be found in those with mild COPD and normal pulmonary artery pressure, suggesting early RV impairment in the course of pulmonary vascular disease.^21^ The association of smoking with worse LV diastolic function and higher LV mass has been previously demonstrated.^14^ LV filling pressures can plausibly affect RV function through backward transmission toward the pulmonary vasculature. Moreover, decrease of coronary blood flow and systemic inflammatory response might also play a role in the relationship between smoking and RV dysfunction.^22 23^ In an experimental model, mice exposed to cigarette smoking showed significantly reduced RV systolic function, as examined by TAPSE, with no changes in either LV parameters or pulmonary vasculature, but with increased cardiac fibroblast proliferation and collagen content in the right ventricle.^24^ Interestingly, nicotine alone reproduced these effects in that study. However, further studies are needed to better elucidate the role of those intermediate factors as significant mediators on the association of tobacco use and RV impairment.

### Limitations

Echocardiography is a useful diagnostic tool for examining RV function.^25^ This study used M-mode-derived TAPSE and tissue Doppler-derived RVS’ as measures of RV systolic function. Both parameters are frequently used in clinical practice, although RV strain from speckle tracking analysis has been considered as a more sensitive index of RV myocardial dysfunction. Unfortunately, RV strain was not available at CARDIA year 25. Unfortunately, in the CARDIA year 25 examination, reproducibility of RV indices was not performed. Nevertheless, echocardiographic exams followed a standardised acquisition protocol, whose manual of operation is available online (https://www.cardia.dopm.uab.edu), and all technicians at each of the four field centres were centrally trained and certified. Also, TAPSE and RVS’ are easy to perform, with very low inter-reader and intrareader observer variability, as demonstrated elsewhere.^26^ The present study has a cross-sectional design; hence, conclusions about temporality or causal associations cannot be made. Pulmonary function data were not available at the time of the year 25 examination. Since pulmonary function was used only as one as multiple confounding variable, it is unlikely that the main associations we observed would have changed if we had used year 25 data on pulmonary function data instead. Although the effect size of the relationship between smoking status and RV function seems not to be high, small effect sizes are not unexpected in relatively healthy young adults, and, importantly, the public health importance is in mitigating the deleterious changes before they become irreversible. However, we recognise that how much these subtle changes in RV function influence risk of heart failure is unknown and should be subject of further research. We recognise that LV dysfunction, pulmonary hypertension, and inflammation are potential mediators in the relationship between smoking status and RV function. However, the cross-sectional design of this present study precludes a reliable mediation analysis, which would be performed in further longitudinal studies.

### Clinical perspectives

Recently, the role of the right ventricle in a spectrum of CVDs has gained renewed attention.^27^ Nevertheless, few studies have addressed the association of smoking and RV adverse remodelling.^13–16 28^ In this large population-based cohort of individuals at middle age, our findings show a relationship between cigarette smoking status and RV systolic and diastolic functions, independent of demographics, traditional cardiovascular risk factors and pulmonary function. Such findings advance knowledge about the harmful effects of tobacco use on the cardiopulmonary unit, and suggest a direct effect of smoking on RV function. Furthermore, our findings support the hypothesis of a potential role of RV impairment for predicting adverse events in smokers.

## Conclusions

In a large multicenter community-based biracial cohort of middle-aged individuals, smoking was related to reduced RV systolic and diastolic functions, independent of demographics, traditional cardiovascular risk factors and pulmonary function.
