# Correction: Association of smoking and right ventricular function in middle age: CARDIA study

**DOI:** 10.1136/openhrt-2020-001270corr1

**Published:** 2020-05-10

Moreira HT, Armstrong AC, Nwabuo CC, *et al*. Association of smoking and right ventricular function in middle age: CARDIA study. *Open Heart* 2020;**7**:e001270.

In the end matter, ‘Provenance and peer review’ statement has been correctly updated as ‘Not commissioned; externally peer reviewed’.

