# Supplementary material for: Association of smoking and right ventricular function in middle age: CARDIA study
Source: Open Heart. 2020 Mar 8;7(1):e001270. doi: 10.1136/openhrt-2020-001270 (PMC7061887; doi:10.1136/openhrt-2020-001270)
Supplement: Supplementary data [file openhrt-2020-001270supp001.pdf]

SUPPLEMENTAL TABLES:

Supplemental table 1. Linear regression analyses for the association of smoking status with RV systolic function assessed by tricuspid annular peak systolic velocity (RVS')

	Univariate			Model 1		
	β	SE	p	β	SE	p
**Smoking Status**						
Never (reference)	ref.	ref.	ref.	ref.	ref.	ref.
Former	-0.033	0.111	0.768	-0.114	0.130	0.383
Current	-0.263	0.122	0.031	-0.343	0.156	0.028
Age (years)	0.006	0.012	0.623	0.014	0.015	0.354
Male (vs. Female)	-0.240	0.089	0.007	-0.400	0.154	0.009
White (vs. Black)	0.086	0.088	0.330	-0.043	0.130	0.741
Education (years)	0.031	0.016	0.057	0.008	0.022	0.704
BMI (Kg/m2)	-0.006	0.006	0.331	0.013	0.009	0.146
SBP (mmHg)	0.003	0.003	0.220	0.004	0.004	0.311
On anti-hypertensive medication	-0.295	0.100	0.003	-0.249	0.135	0.066
Total cholesterol (mg/dL)	0.001	0.001	0.896	-0.003	0.002	0.050
HDL-cholesterol (m/dL)	0.012	0.002	<0.001	0.021	0.004	<0.001
On lipid-lowering medication	-0.177	0.122	0.146	-0.103	0.157	0.513
Diabetes Mellitus	-0.221	0.136	0.105	0.067	0.177	0.705
Alcohol consumption (mL/day)	0.005	0.002	0.005	0.006	0.002	0.023
FEV1 (L)	0.224	0.061	<0.001	0.183	0.099	0.064
Left ventricular ejection fraction (%)	0.011	0.001	0.075	0.002	0.007	0.833
E/e' ratio	0.028	0.019	0.139	0.067	0.024	0.005
Coronary artery calcium score (AU)	-0.001	0.001	0.259	-0.001	0.001	0.001

β = coefficient; FEV1 = forced expiratory volume at the first second; AU = Agatston unit
Model 1 = adjusted for all covariates.

Supplemental table 2. Linear regression analyses for the association of smoking status with RV systolic function assessed by tricuspid annular plane systolic excursion (TAPSE)

	Univariate			Model 1		
	β	SE	p	β	SE	p
**Smoking Status**						
Never (reference)	ref.	ref.	ref.	ref.	ref.	ref.
Former	0.005	0.023	0.819	-0.023	0.026	0.376
Current	-0.115	0.025	<0.001	-0.082	0.031	0.008
Age (years)	0.006	0.002	0.023	0.006	0.003	0.063
Male (vs. Female)	0.015	0.018	0.413	0.076	0.028	0.014
White (vs. Black)	0.105	0.018	<0.001	0.071	0.031	0.001
Education (years)	0.008	0.003	0.013	-0.007	0.004	0.118
BMI (Kg/m2)	0.006	0.001	<0.001	0.009	0.002	<0.001
SBP (mmHg)	0.001	0.001	0.928	0.001	0.001	0.922
On anti-hypertensive medication	-0.071	0.020	<0.001	-0.065	0.027	0.018
Total cholesterol (mg/dL)	-0.001	0.000	0.003	-0.001	0.001	<0.001
HDL-cholesterol (m/dL)	0.001	0.001	0.862	0.001	0.001	0.033
On lipid-lowering medication	-0.036	0.025	0.148	-0.034	0.031	0.290
Diabetes Mellitus	-0.066	0.028	0.017	-0.047	0.036	0.187
Alcohol consumption (mL/day)	0.001	0.001	0.405	0.001	0.001	0.032
FEV1 (L)	0.224	0.061	<0.001	0.098	0.020	<0.001
Left ventricular ejection fraction (%)	0.003	0.001	0.008	0.003	0.002	0.066
E/e’ ratio	0.013	0.004	0.001	0.018	0.005	< 0.001
Coronary artery calcium score (AU)	-0.001	0.001	0.004	-0.001	0.001	0.005

β = coefficient; FEV1 = forced expiratory volume at the first second; AU = Agatston unit
Model 1 = adjusted for all covariates.

Supplemental table 3. Linear regression analyses for the association of smoking status with RV diastolic function assessed by early diastolic tricuspid annular tissue velocity (RVE')

	Univariate			Model 1		
	β	SE	p	β	SE	p
**Smoking Status**						
Never (reference)	ref.	ref.	ref.	ref.	ref.	ref.
Former	-0.371	0.140	0.008	-0.414	0.162	0.011
Current	-0.847	0.155	<0.001	-0.715	0.195	<0.001
Age (years)	-0.032	0.015	0.036	-0.018	0.019	0.351
Male (vs. Female)	0.750	0.112	<0.001	0.404	0.191	0.035
White (vs. Black)	0.268	0.112	0.017	-0.187	0.162	0.248
Education (years)	0.114	0.021	<0.001	0.053	0.027	0.048
BMI (Kg/m2)	-0.051	0.008	<0.001	-0.028	0.011	0.014
SBP (mmHg)	-0.018	0.003	<0.001	-0.007	0.005	0.136
On anti-hypertensive medication	-0.809	0.126	<0.001	-0.388	0.169	0.021
Total cholesterol (mg/dL)	-0.002	0.002	0.269	-0.004	0.002	0.037
HDL-cholesterol (m/dL)	0.020	0.003	<0.001	0.010	0.004	0.018
On lipid-lowering medication	-0.534	0.154	0.001	0.003	0.196	0.988
Diabetes Mellitus	-0.930	0.172	<0.001	-0.244	0.221	0.270
Alcohol consumption (mL/day)	-0.004	0.002	0.089	0.002	0.003	0.545
FEV1 (L)	-0.093	0.076	0.225	0.046	0.123	0.710
Left ventricular ejection fraction (%)	0.040	0.008	<0.001	0.025	0.009	0.006
E/e' ratio	-0.011	0.024	<0.001	-0.046	0.030	0.126
Coronary artery calcium score (AU)	-0.002	0.001	<0.001	-0.001	0.001	<0.001

β = coefficient; FEV1 = forced expiratory volume at the first second; AU = Agatston unit
Model 1 = adjusted for all covariates.

Supplemental table 4. Linear regression models for the association of smoking and RV systolic function assessed by tricuspid annular peak systolic velocity (RVS') in current smokers

	Univariate			Model 1		
	β-Coefficient	SE	p	β-Coefficient	SE	p
**Cigarettes smoked per day**						
1 - 6	ref.	ref.	ref.	ref.	ref.	ref.
7 – 14	-0.121	0.265	0.647	0.106	0.303	0.727
≥ 15	0.028	0.259	0.915	0.083	0.317	0.793
**Years smoking regularly**						
< 26	ref.	ref.	ref.	ref.	ref.	ref.
26-32	0.334	0.262	0.204	0.053	0.305	0.862
≥ 33	0.173	0.260	0.507	-0.393	0.334	0.240
**Cigarette pack-years**						
< 4.7	ref.	ref.	ref.	ref.	ref.	ref.
4.7-11.7	0.024	0.264	0.929	0.212	0.302	0.482
> 11.7	0.084	0.266	0.752	-0.078	0.323	0.810

Model 1 = adjusted for age, sex, race, years of education, body mass index, systolic blood pressure, use of anti-hypertensive medication, total cholesterol, HDL-cholesterol, use of lipid lowering medication, diabetes mellitus, alcohol consumption, pulmonary function, left ventricular systolic and diastolic function, and coronary artery calcium score.

Supplemental table 5. Linear regression models for the association of smoking and RV systolic function assessed by tricuspid annular plane systolic excursion (TAPSE) in current smokers

	Univariate			Model 1		
	β-Coefficient	SE	p	β-Coefficient	SE	p
**Cigarettes smoked per day**						
1 - 6	ref.	ref.	ref.	ref.	ref.	ref.
7 – 14	-0.049	0.052	0.350	0.023	0.062	0.715
≥ 15	0.005	0.051	0.923	0.032	0.065	0.625
**Years smoking regularly**						
< 26	ref.	ref.	ref.	ref.	ref.	ref.
26-32	-0.052	0.052	0.313	-0.071	0.063	0.265
≥ 33	-0.116	0.051	**0.024**	-0.089	0.068	0.193
**Cigarette pack-years**						
< 4.7	ref.	ref.	ref.	ref.	ref.	ref.
4.7-11.7	0.005	0.052	0.917	0.074	0.062	0.237
> 11.7	0.030	0.052	0.565	0.060	0.066	0.366

Model 1 = adjusted for age, sex, race, years of education, body mass index, systolic blood pressure, use of anti-hypertensive medication, total cholesterol, HDL-cholesterol, use of lipid lowering medication, diabetes mellitus, alcohol consumption, pulmonary function, left ventricular systolic and diastolic function, and coronary artery calcium score.

Supplemental table 6. Linear regression model for the association of smoking and RV diastolic function assessed by early diastolic tricuspid annular tissue velocity (RVE') in current smokers

	**Univariate**			**Model 1**		
	β-Coefficient	SE	p	β-Coefficient	SE	p
**Cigarettes smoked per day**						
1 - 6	ref.	ref.	ref.	ref.	ref.	ref.
7 – 14	-0.379	0.317	0.232	0.084	0.379	0.825
≥ 15	-0.608	0.309	0.049	0.204	0.395	0.606
**Years smoking regularly**						
< 26	ref.	ref.	ref.	ref.	ref.	ref.
26-32	0.226	0.313	0.471	0.338	0.381	0.375
≥ 33	-0.439	0.309	0.156	0.030	0.413	0.943
**Cigarette pack-years**						
< 4.7	ref.	ref.	ref.	ref.	ref.	ref.
4.7-11.7	-0.232	0.316	0.464	0.175	0.377	0.642
> 11.7	-0.546	0.317	0.086	0.251	0.401	0.531

Model 1 = adjusted for age, sex, race, years of education, body mass index, systolic blood pressure, use of anti-hypertensive medication, total cholesterol, HDL-cholesterol, use of lipid lowering medication, diabetes mellitus, alcohol consumption, and pulmonary function, left ventricular systolic and diastolic function, and coronary artery calcium score.
